# Supplementary material for: Pyruvate Dehydrogenase Kinase 1 inhibition mediated oxidative phosphorylation enhancement in cartilage promotes osteoarthritis progression
Source: BMC Musculoskelet Disord. 2023 Jul 20;24:597. doi: 10.1186/s12891-023-06585-6 (PMC10357736; doi:10.1186/s12891-023-06585-6)
Supplement: Supplementary file 1 — Additional file 1. [file 12891_2023_6585_MOESM1_ESM.zip › KEGG.Gsea.1653622432464/KEGG_NITROGEN_METABOLISM.html]

Details for gene set KEGG\_NITROGEN\_METABOLISM[GSEA]

|  || Dataset | 55457\_collapsed\_to\_symbols.cls.cls #OA\_versus\_normal.cls.cls #OA\_versus\_normal\_repos |
| Phenotype | cls.cls#OA\_versus\_normal\_repos |
| Upregulated in class | normal |
| GeneSet | KEGG\_NITROGEN\_METABOLISM |
| Enrichment Score (ES) | -0.3306679 |
| Normalized Enrichment Score (NES) | -1.038155 |
| Nominal p-value | 0.3939394 |
| FDR q-value | 0.5862714 |
| FWER p-Value | 1.0 |
Table: GSEA Results Summary

  

Fig 1: Enrichment plot: KEGG\_NITROGEN\_METABOLISM      
 Profile of the Running ES Score & Positions of GeneSet Members on the Rank Ordered List

  

| SYMBOL | TITLE | RANK IN GENE LIST | RANK METRIC SCORE | RUNNING ES | CORE ENRICHMENT || 1 | GLUD1 | glutamate dehydrogenase 1 [Source:HGNC Symbol;Acc:HGNC:4335] | 1279 | 0.462 | -0.0266 | No |
| 2 | GLUD2 | glutamate dehydrogenase 2 [Source:HGNC Symbol;Acc:HGNC:4336] | 1554 | 0.420 | 0.0182 | No |
| 3 | CPS1 | carbamoyl-phosphate synthase 1 [Source:HGNC Symbol;Acc:HGNC:2323] | 2178 | 0.341 | 0.0235 | No |
| 4 | CA3 | carbonic anhydrase 3 [Source:HGNC Symbol;Acc:HGNC:1374] | 3671 | 0.188 | -0.0627 | No |
| 5 | CA5B | carbonic anhydrase 5B [Source:HGNC Symbol;Acc:HGNC:1378] | 4333 | 0.125 | -0.0943 | No |
| 6 | CA14 | carbonic anhydrase 14 [Source:HGNC Symbol;Acc:HGNC:1372] | 4753 | 0.091 | -0.1125 | No |
| 7 | CA9 | carbonic anhydrase 9 [Source:HGNC Symbol;Acc:HGNC:1383] | 6142 | -0.015 | -0.2178 | No |
| 8 | CA8 | carbonic anhydrase 8 [Source:HGNC Symbol;Acc:HGNC:1382] | 6397 | -0.035 | -0.2320 | No |
| 9 | CA7 | carbonic anhydrase 7 [Source:HGNC Symbol;Acc:HGNC:1381] | 7210 | -0.097 | -0.2798 | No |
| 10 | CA12 | carbonic anhydrase 12 [Source:HGNC Symbol;Acc:HGNC:1371] | 7520 | -0.120 | -0.2849 | No |
| 11 | CA1 | carbonic anhydrase 1 [Source:HGNC Symbol;Acc:HGNC:1368] | 7585 | -0.126 | -0.2700 | No |
| 12 | CA2 | carbonic anhydrase 2 [Source:HGNC Symbol;Acc:HGNC:1373] | 7708 | -0.135 | -0.2583 | No |
| 13 | GLS | glutaminase [Source:HGNC Symbol;Acc:HGNC:4331] | 8423 | -0.187 | -0.2843 | No |
| 14 | AMT | aminomethyltransferase [Source:HGNC Symbol;Acc:HGNC:473] | 9021 | -0.234 | -0.2939 | Yes |
| 15 | HAL | histidine ammonia-lyase [Source:HGNC Symbol;Acc:HGNC:4806] | 9053 | -0.236 | -0.2592 | Yes |
| 16 | CA6 | carbonic anhydrase 6 [Source:HGNC Symbol;Acc:HGNC:1380] | 9260 | -0.253 | -0.2354 | Yes |
| 17 | CA4 | carbonic anhydrase 4 [Source:HGNC Symbol;Acc:HGNC:1375] | 10464 | -0.369 | -0.2707 | Yes |
| 18 | CTH | cystathionine gamma-lyase [Source:HGNC Symbol;Acc:HGNC:2501] | 11108 | -0.444 | -0.2506 | Yes |
| 19 | GLS2 | glutaminase 2 [Source:HGNC Symbol;Acc:HGNC:29570] | 11547 | -0.507 | -0.2048 | Yes |
| 20 | ASNS | asparagine synthetase (glutamine-hydrolyzing) [Source:HGNC Symbol;Acc:HGNC:753] | 11913 | -0.574 | -0.1427 | Yes |
| 21 | CA5A | carbonic anhydrase 5A [Source:HGNC Symbol;Acc:HGNC:1377] | 12145 | -0.630 | -0.0614 | Yes |
| 22 | GLUL | glutamate-ammonia ligase [Source:HGNC Symbol;Acc:HGNC:4341] | 12527 | -0.762 | 0.0290 | Yes |
Table: GSEA details [plain text format]

  

Fig 2: KEGG\_NITROGEN\_METABOLISM      
 Blue-Pink O' Gram in the Space of the Analyzed GeneSet

  

Fig 3: KEGG\_NITROGEN\_METABOLISM: Random ES distribution      
 Gene set null distribution of ES for **KEGG\_NITROGEN\_METABOLISM**
